# Supplementary material for: Continuous estimation of power system inertia using convolutional neural networks
Source: Nat Commun. 2023 Jul 24;14:4440. doi: 10.1038/s41467-023-40192-2 (PMC10366096; doi:10.1038/s41467-023-40192-2)
Supplement: Supplementary file 1 — Supplementary Information [file 41467_2023_40192_MOESM1_ESM.pdf]

Supplementary information
Continuous Estimation of Power System Inertia Using Convolutional Neural Networks

Daniele Linaro[1,*], Federico Bizzarri[1,2], Davide del Giudice[1], Cosimo Pisani[3], Giorgio M. Giannuzzi[3], Samuele Grillo[1], and Angelo Brambilla[1]

[1]DEIB, Politecnico di Milano, P.zza Leonardo da Vinci 32, Milano, 20133, Italy
[2]ARCES, University of Bologna, Bologna, 41026, Italy
[3]Terna Rete Italia S.p.A., V.le Egidio Galbani, 70, Rome, 00156, Italy
[*]Corresponding author: daniele.linaro@polimi.it

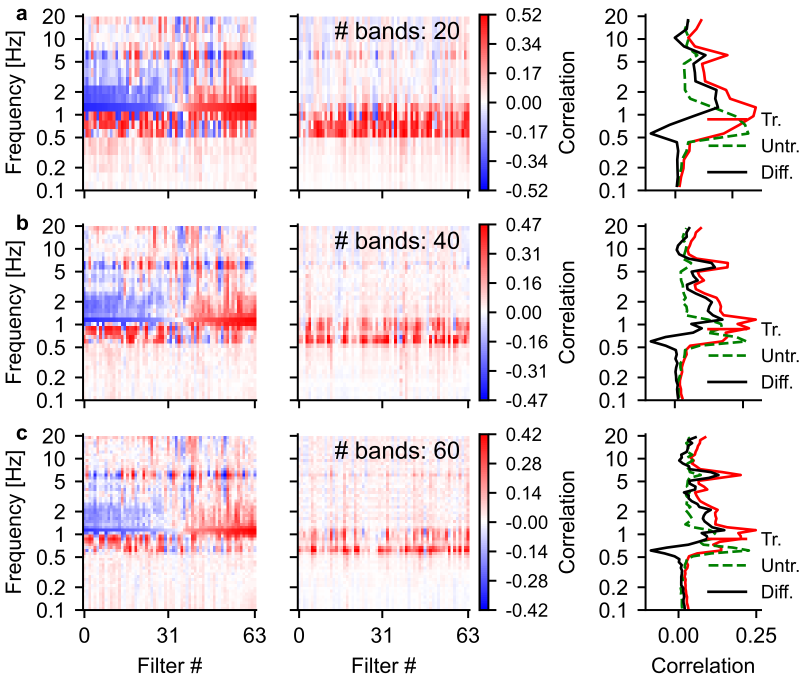

Figure S1: **Correlation magnitude depends on the number of subdivisions of the frequency interval.** **a** Left and center, correlation maps for the trained and untrained networks when the interval $(0.1, 20)$ Hz is subdivided into 20 (logarithmically) equally spaced bins. Right, mean absolute correlation computed over all filters for the trained (red trace) and untrained (dashed green trace) networks, and the difference between the two (black trace). **b,c** Same as **a**, but for 40 and 60 subdivisions, respectively: notice the different scales used for the correlation values.

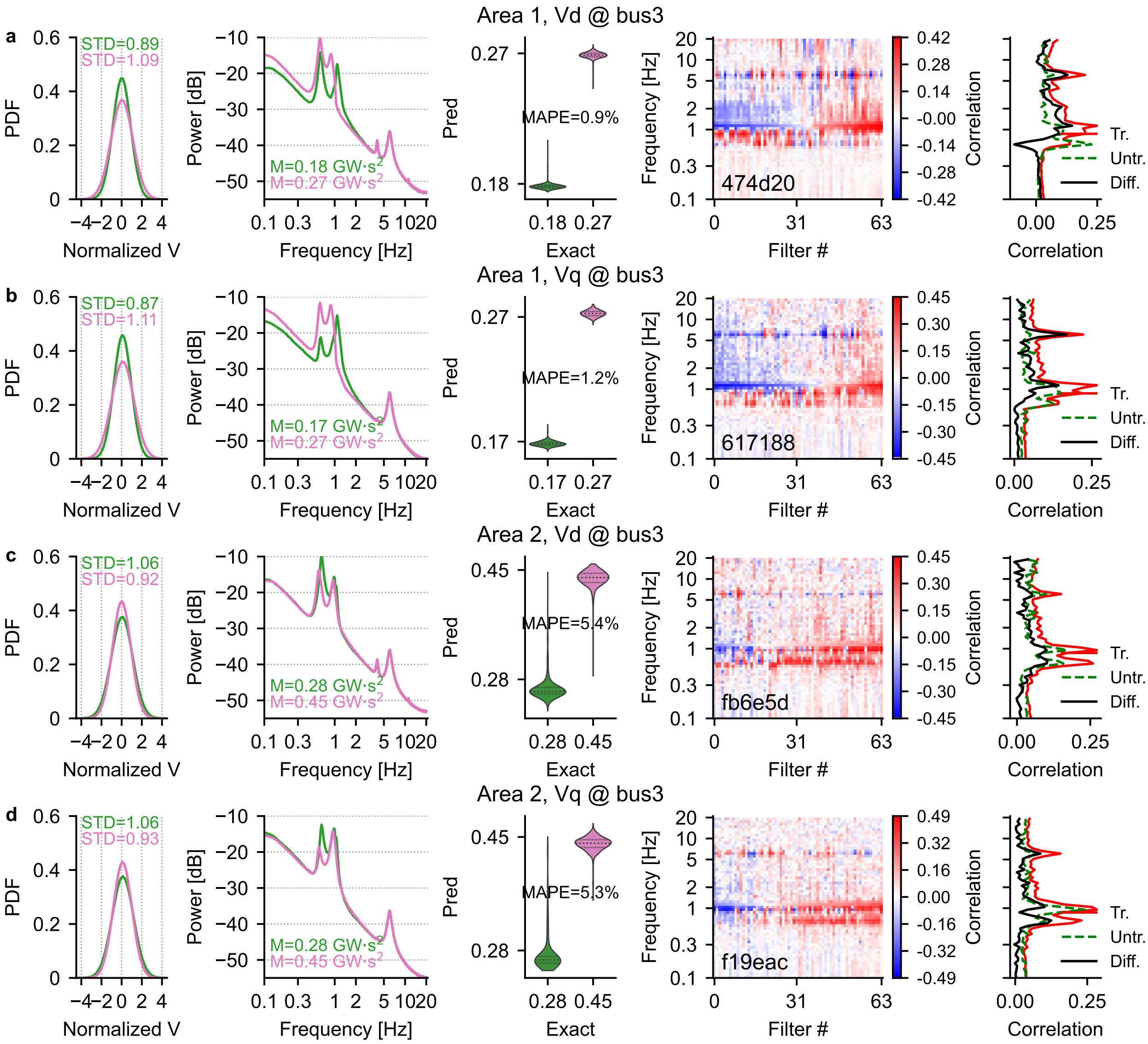

Figure S2: **Choice of bus and area affects the performance of the CNN.** In each panel, green and magenta traces refer to low and high area momentum, respectively. The specific values of momentum for each condition are indicated in the panels of the second column. **a** Panels from left: (1) distribution of the direct voltage at bus 3, with low area momentum leading to a smaller standard deviation. (2) Spectra of the direct voltage at bus 3. (3) Violin plot of the momentum values predicted by a CNN trained on the direct voltage at bus 3 (MAPE on the test set indicated in the figure). (4) Correlation map for the trained network (experiment ID indicated in the figure). (5) Mean absolute correlation over all filters for the trained (red trace) and untrained (dashed green trace) networks, and the difference between the two (black trace). **b** Same as **a**, but for the quadrature voltage at bus 3: only a limited increase in MAPE is apparent, while the correlation map presents a strikingly similar structure to the one in **a**. **c,d** Same as **a,b**, but for direct and quadrature voltages recorded at bus 3 when the momentum of area 2 is changed.

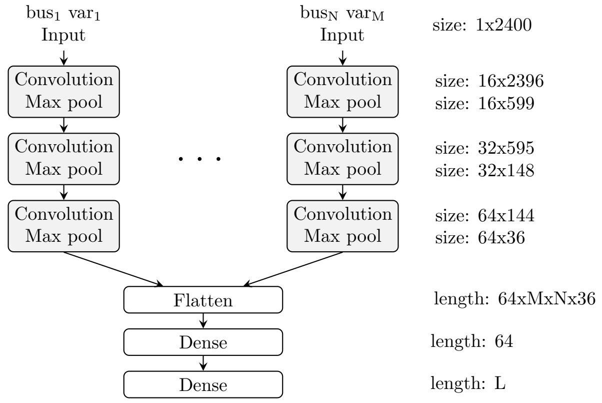

Figure S3: **Architecture of the CNN.** The three gray blocks containing one convolution and one max pooling layer are replicated for each of the electrical variables used as inputs to the network. The column on the right indicates the shape of the output of each layer: "size" and "length" indicate that the output is a matrix or a vector, respectively.

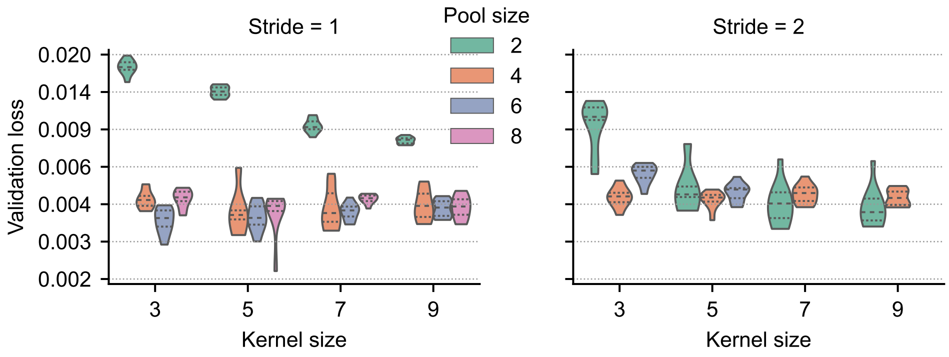

Figure S4: **Hyperparameters impact on CNN accuracy**. Left, validation loss as a function of kernel size and max pooling size when kernel stride was fixed to 1. Violin plots indicate the distribution of validation losses for $n = 10$ networks trained in each condition. Right, same as left panel, but for kernel stride equal to 2. In this case, not all combinations of kernel size and max pooling size considered in the left panel are feasible, as detailed in the Methods.

Table S1: Inertia constants and corresponding area momenta for training the simple CNN.

Parameter set	H_{G_2} [s]	H_{G_3} [s]	Momentum [GWs^2]	
■	3.33	3.47	0.171	
■	3.53	3.47	0.176	0.176
■	3.33	3.67	0.175	
■	3.53	3.67	0.181	
■	5.13	5.27	0.261	
■	5.33	5.27	0.266	0.266
■	5.13	5.47	0.265	
■	5.33	5.47	0.271	

Table S2: Momentum predictions for CNNs trained without and with variable compensator's inertia.

				Momentum [GWs^2]		
Parameter set	H_{G_2} [s]	H_{G_3} [s]	H_C [s]	exact	w/o comp	w/ comp
■	3.497	3.637	0.1	0.179	0.180 ± 0.005	0.178 ± 0.002
■	3.863	4.003	0.1	0.197	0.199 ± 0.012	0.192 ± 0.013
■	3.463	3.603	6.1	0.197	0.184 ± 0.003	0.197 ± 0.002
■	5.297	5.437	0.1	0.269	0.268 ± 0.006	0.267 ± 0.002

Table S3: Inertia constants and corresponding area momentum for the simulations shown in Fig. 7. The labels in the right-most column correspond to the panels of Fig. 7.

Sim. time [min]	H_{G_2} [s]	H_{G_3} [s]	H_{C_1} [s]	Momentum [GWs^2]	
$(0, 60)$	4.33	4.47	0.1	0.2206	
$(60, 120)$	3.83	3.97	0.1	0.1956	A
$(120, 180)$	4.83	4.97	0.1	0.2456	
$(0, 60)$	4.33	4.47	0.1	0.2206	
$(60, 120)$	3.83	4.9075	0.1	0.2206	B
$(120, 180)$	4.9014	3.97	0.1	0.2206	
$(0, 60)$	4.33	4.47	0.1	0.2206	
$(60, 120)$	4.2415	4.8474	0.1	0.2286	C
$(120, 180)$	4.3964	5.0244	0.1	0.2369	
$(0, 60)$	4.33	4.47	0.1	0.2206	
$(60, 120)$	4.33	4.47	2.5	0.2286	D
$(120, 180)$	4.33	4.47	5.0	0.2369	

Table S4: Comparison of CNN performance with other ML models. The MAPE on the test set is presented as mean ± SEM over $N = 10$ repetitions for those models that use a stochastic training algorithm. Parameter counts include only those parameters that are modified during training: see the Methods for the values of each model's hyperparameters.

Model	MAPE [%]	N	Parameter count	Training duration
CNN	1.07 ± 0.08	10	160 577	292 s
SVR	5.59	1	∼ 24 000 000	1144 s
MLP	6.07 ± 0.06	10	160 935	141 s
Nearest neighbors	9.30	1	0	22 h
Kernel ridge	10.82	1	10 000	4 s
Random forest	14.95 ± 0.02	10	173 352	283 s

Table S5: Hyperparameters impact on CNN accuracy. Rows highlighted in green correspond to optimal hyperparameters combinations, while the row highlighted in blue indicates the hyperparameters combination used throughout this work.

Kernel size	Kernel stride	Pooling size	Output size	Median val. loss (n=10)
3	1	2	298	0.01759
3	2	2	37	0.01056
5	1	2	296	0.01369
5	2	2	36	0.00477
7	1	2	294	0.00950
7	2	2	35	0.00434
9	1	2	293	0.00835
9	2	2	35	0.00397
3	1	4	36	0.00450
3	2	4	4	0.00467
5	1	4	36	0.00386
5	2	4	4	0.00461
7	1	4	35	0.00394
7	2	4	3	0.00483
9	1	4	34	0.00424
9	2	4	3	0.00459
3	1	6	10	0.00374
3	2	6	1	0.00608
5	1	6	10	0.00374
5	2	6	1	0.00501
7	1	6	9	0.00404
9	1	6	9	0.00416
3	1	8	4	0.00462
5	1	8	4	0.00423
7	1	8	3	0.00458
9	1	8	3	0.00421

Table S6: Range of momenta for the generation of the data used in this study.

	Training set	Test set	Validation set
H_{G_2}[s]	(3.33,5.33)	(3.463,5.463)	(3.597,5.597)
H_{G_3}[s]	(3.47,5.47)	(3.603,5.603)	(3.737,5.737)
Min. M [GWs^2]	0.1706	0.1772	0.1839
Max. M [GWs^2]	0.2706	0.2772	0.2839
Steps	6×6	6×6	6×6
Duration per step [s]	300×10^3	30×10^3	30×10^3
